# Promoting Intergenerational Engagement Within the College Classroom: Faculty Training Needs

**DOI:** 10.1093/geroni/igab046.1413

**Published:** 2021-12-17

**Authors:** Afeez Hazzan, Kristin Heffernan, Jason Dauenhauer

**Affiliations:** 1 State University of New York at Brockport, Brockport, New York, United States; 2 State University of New York, Brockport, State University of New York, Brockport, New York, United States; 3 State University of New York, Brockport, Brockport, New York, United States

## Abstract

As population aging accelerates worldwide, institutions of higher education are increasing efforts to focus on ways to meet the growing needs of older adult learners. Many institutions are addressing these needs by joining the Age-Friendly University (AFU) Global Network. Affiliated institutions are required to promote intergenerational learning to facilitate the reciprocal sharing of expertise between learners of all ages, including older adults. However, these institutions will need to provide instructors with the training to ensure that intergenerational engagement is being actively fostered in the classrooms. In this study, we examine the perspectives of faculty members who have opened their classrooms to older adult auditors. The research question was: What types of training do faculty recommend to promote intergenerational engagement in the classroom? In-depth face to face interviews were conducted with 27 faculty members. Qualitative content analysis of the data yielded the following four themes: 1) Provide accessible training to teach faculty their role 2) Educate faculty about the importance of becoming aware of generational time periods/context 3) Learn to approach auditors with a mindset that they are adults and have had careers/experiences, and 4) Train faculty on how to foster discussion. Overall, these findings point to a need for training that focus on intergenerational curricular design and multigenerational classroom management.

